# Risk factors for surgical site infections in obstetrics: a retrospective study in an Ethiopian referral hospital

**DOI:** 10.1186/s13037-017-0138-9

**Published:** 2017-09-19

**Authors:** Teshager Mamo, Tilaye Workneh Abebe, Tesfaye Yitna Chichiabellu, Antehun Alemayehu Anjulo

**Affiliations:** 1 Department of General Surgery at Bale Ginnir Hospital, Ginnir, Ethiopia; 2 Department of Public Health, Adama Hospital Medical College, Adama, Ethiopia; 3 Department of Nursing, College of Health Science and Medicine, Wolaita Sodo University, Wolaita Sodo, Ethiopia; 4 Department of Medical Laboratory, School of Medicine, College of Health sciences and Medicine, Wolaita Sodo University, P.O. Box: 138, Wolaita Sodo, Ethiopia

**Keywords:** Surgical site infection, Risk factors

## Abstract

**Background:**

Pregnant women are at risk of infection during labor and delivery. Infection in obstetrics accounts for the second most common cause of maternal mortality next to post partum hemorrhage. Knowing the prevalence and associated risk factors would help to undertake optimal precautions and standard surgical techniques to reduce surgical site infection which poses increased hospital cost and total hospital stay of the patients.

**Method:**

Facility based retrospective observational study design was carried out purposively to assess the prevalence of surgical site infections and associated risk factors among mothers who had delivery related surgery at obstetric ward of Assela teaching referral hospital from April, 23, 2015 to September 5, 2015. A total of 384 women who had surgery for delivery were included in the study. The risk associated with SSIs was assessed by multivariate regression logistic analysis.

**Results:**

The age of the women ranged from 17 to 40 years with the mean age of 26 (±5) years. The rate of surgical site infection was 9.4%(36/384). The risk factors for surgical site infection were age less than 19 (OR = 3.5, 95%CI 1.17–10.01), preterm gestation age (OR = 4.225 95%CI 1.254–14.238), duration of labor ≥24 h (OR = 2.219 95%CI1.054–4.670), duration of rupture of membrane ≥12 h (OR = 5.99, 95% CI2.75–13.02),chorioamnionitis (OR = 9.743, 95%CI 3.077–30.848), vertical skin incision(OR = 4,95%CI 1.709–13.322), pre operation Hematocrit (OR = 6.4,95%CI1.021–40.137),perioperative blood transfusion(OR = 6.75,95%CI 2.47,18.49), abdominal hysterectomy(OR = 7.9,95%CI1.698–36.960), and diabetic mellitus (OR = 3.7,95%CI 1.112–12.519).

**Conclusions:**

Obstetric ward of Assela teaching referral hospital are encouraged to use properly WHO surgical safety checklist and examine how to sensibly integrate these essential safety steps into their normal operative workflow. Prophylactic antibiotic administration should be provided within one hour before the surgical incision or within two hours if the patient is receiving vancomycin or floroquinolones.

## Background

Pregnant women are at risk of infection during labor and delivery; most infections of the female pelvic organs occur when normal flora of the female genital or gastrointestinal tract contaminate the normally sterile amniotic fluid and uterus [1]. Infection in obstetrics accounts for the second most common cause of maternal mortality next to post partum hemorrhage [2].

Centers for Disease Control and Prevention’s (CDC’s) and National Healthcare Safety Network

(NHSN) categorized Surgical Site Infections (SSIs) in to 3 groups. According to this classification Organ/space SSIs involve any part of the anatomy and that must develop within 30 days after procedures (e.g. organ/space) but does not include incision body wall layers that was opened or manipulated during an operation. Incision SSIs involving only skin and subcutaneous tissue are grouped as superficial incision SSIs and incision involving the deeper soft tissue classified as deep incision SSI [3–6].

SSIs are the most common nosocomial infections, accounting for 38% of hospital acquired infections [7] and each SSI demanding the cost of $2739 in USA [1, 8]. Infections prolonged hospitalization from 1.5 to 16.6 days [9–12]. Studies showed that the prevalence of SSI after cesarean section varied from 3% to15%, depending on the surveillance methods used to identify infections, the patient population, and the use of antibiotic prophylaxis [13]. It is highly prevalent and more serious in developing countries; especially in sub-Saharan Africa; twice or three times higher than developed countries [1, 14, 15].

When we see the distribution of SSIs in Africa; the cumulative incidence in Algerian was 11.9% in 2001, 9.4% among women who underwent caesarean section in Ugandan, 23.6 per 100 operations in Nigeria [8], In Ethiopia, there was only one study done in Jimma University Specialized Hospital, Southwest Ethiopia. According to this study the prevalence of SSI following cesarean Section accounted for 66 (75.0%) [16].

According to previous report, maternal morbidity related to infections after cesarean section was eight-fold higher than that of vaginal delivery [10–12]. Cesarean section is one of the most commonly performed obstetrical surgical procedures in Assela teaching hospital, the rate of cesarean section has increased from approximately 3% in the 1994 to more than 15% recently. Knowing the prevalence of the problem and associated risk factors would help to undertake optimal precautions and standard surgical techniques to reduce SSI which poses increased hospital cost and total hospital stay of the patients. This study intended to assess the prevalence of SSI and associated risk factors.

## Methods

### Study area

The study was conducted in Assela town. Assela town is located in Oromia region, 170 km far from Addis Ababa. The hospital has been serving 3.5 million populations and in Arsi and the nearby zones; have 331 beds in total. Department of Obstetrics and Gynecology has two wards. Obstetrics ward has 49 bed and Gyn. Ward 29 bed. MCH and family planning clinic, and two Gynecologic OPD. It has three consultant Obstetricians & Gynecologists, six physicians, seventeen midwives and eight clinical nurses. Reviewing the registration log book, there were 3618 obstetric cases among this 945 cesarean section and 1 destructive delivery under direct vision in the last one year.

### Study design and period

Facility based retrospective observational study design was carried out purposively to assess the prevalence of surgical site infections and associated risk factors among mothers who had delivery related surgery at obstetric ward of Assela teaching referral hospital from April, 23, 2015 to September 5, 2015 till to reach the calculated sample size.

### Population

#### Source population

All women who had major surgery related delivery at Assela teaching hospital.

#### Study population

All women who had major surgery related delivery in obstetrics ward at Assela teaching hospital during the study period.

#### Inclusion criteria

- All 384 women who had surgery related to delivery and who developed surgical site infections in obstetrics ward at Assela teaching hospital

#### Exclusive criteria

- Patients who died before the third post operative days were excluded, because SSIs cannot be diagnosed before three days of operative procedure.
- Women referred from other health care facilities for the diagnosis of SSIs
- Infection associate with minor procedures like episiotomy and stitch site infection

### Sample size and sampling procedures

A single population proportion formula was used to attain 95% confidence interval, 50% prevalence of SSIs and 5% margin of error were taken. The calculated sample size was 384. Then, study subjects were selected using consecutive sampling technique.

### Data collection instrument and procedure

A semi-structured interviewer administered data collection format was used to collect data for those who developed surgical site infections both from the records and from the study participants. Questionnaire was adopted from similar study and modified to the context of study area after reviewing relevant literatures [16]. It includes basic demographic details and risk factors associated with surgical site infection. Checklist was used to collect data from the medical records. Laboratory reporting format was used for hemoglobin measurement.

### Data processing and analysis

After individual data were scrutinized thoroughly for completeness, coding was done accordingly and the data were subsequently fed in to SPSS version 20.0 for analysis. After descriptive analysis was done; association between dependent variable and the risk factors was determined by comparing each group separately with univariate analysis. On the basis of the results in the univariate analysis, statistically significant variables were included in the multivariate model. Multivariable regression was used to adjust or control the possible confounding factors and to identify risk factors of SSIs. Odd ratio was used with 95% confidence interval to identify factor significant associated with SSI. The cut point for Statistical significance was *P* < 0.05.

## Result

### Socio-demographic characteristics of study population

Overall, 1399 mothers visited the hospital related to delivery and 390 underwent operation during the study period. Three hundred eighty four patients were enrolled in the study (5 patients refused to participate, and 1 patient died immediately after operation). The age of the women ranged from 15 to 40 years with the mean age of 26 (±5) years. Majority of women 309(80.5%) were in the age group of 20–34 years. About 218(56.8%) were resident of rural area and the rest 166(43.2%) were from urban area. Regarding ethnicity; 219(67.4%) were Oromo. Majority of women were Muslim in religion; 184(47.9%) followed by orthodox Christian and protestant. With regard to job, 225(58.6%) of the study participants were House wife. Concerning the educational status of the participants; majority 77(20%) were illiterate. In this study 381(99.7%) were married in terms of marital status. Majority of the study participant family the monthly income was <1000birr or 48.5 dollars; which accounts 175(45.6%) (Table 1).

**Table 1:** Socio-demographic characteristics’ of women having surgery for delivery in obstetrics of Assela Teaching Hospital, April, 23, 2015 to September 5, 2015

Variable		Frequency	Percent
Age	≤ 19	23	13.9
	20–34	309	63.9
	≥35	52	22.2
Address	Urban	166	47.2
	Rural	218	52.8
Ethnicity	Oromo	259	67.4
	Amhara	62	16.1
	Tigrie	32	8.3
	Guragie	20	5.2
	Other	11	2.9
Religion	Orthodox Christian	136	35.4
	Protestant	61	15.9
	Muslim	184	47.9
	Other	3	0.8
Occupation	House wife	225	58.6
	Civil servant	100	26
	Farmer	3	8
	Merchant	56	14.6
Educational Status	Illiterate	77	20.1
	Read &Write Only	54	13.8
	Grade 1–8	68	17.7
	Grade 9–12	98	25.5
	Above grade 12	88	22.9
Marital Status	Married	383	99.7
	Divorced	1	0.3
Average monthly income of Family	Less than 1000	175	45.6
	1001–3999	107	27.9
	Greater than 4000	102	26.6

### Types of surgical procedure

A total of 370(88.9%) underwent cesarean section. However 330 out of 370(89.6%) were emergency cesarean section and the remaining 40 (10.4%) were underwent elective cesarean sections (Table 2). Moreover, 9(8.3%) underwent abdominal hysterectomy and only 5(2.8%) underwent uterine repair. Majority of the operations were emergency cases.

**Table 2:** Indications for cesarean section in Assilla Teaching Hospital; April, 23, 2015 to September 5, 2015

Indication	Frequency	Percent
NRFHBP	121	31.5
CPD	42	10.9
Protracted /arrest of cervical dilatation	31	8.1
Previous two cesarean section scar	21	5.5
Failed vacuum/forceps	20	5.2
Macrosomia	20	5.2
Failed induction/augmentation	18	4.7
Other ^a^	111	28.9
Total	384	100

^a^-placenta previa, twin A breech, declined VBACS, severe pre-eclamsia, poor BPP

### Magnitude of SSI

A total of 36 out of 384 (9.4%) developed SSIs following major surgery related to delivery in obstetrics ward at Assela teaching referral hospital. SSIs were prevalent among women underwent cesarean section; its prevalence was 88.9% followed by women underwent abdominal hysterectomy and uterine repair; which account 8.3% and 2.8% respectively. SSIs were more frequent among women underwent emergency cesarean section (86.1%) than elective cesarean section (13.9%). From women who developed SSIs; 61.1% knew their infection after discharge. The mean day in order to follow up the development postoperative SSIs and the mean number of additional postoperative day of hospital stay due to SSIs were 7.7 (4–18 days) and 13.8(5- 62 days) respectively (Table 3).

**Table 3:** Outcomes of mothers with SSIs following surgery for delivery in Obstetrics of Assela Teaching Hospital, April, 23, 2015 to September 5, 2015

Variables	Category	Frequency(*N* = 36)	Percent
The time surgical site infection detected	Before discharge	14	38.9
	After discharge	22	61.1
Types of surgical site infections detected	Superficial	11	30.5
	Deep	23	63.8
	Organ /space	2	5.7
Postoperative day SSIs detected	≤ 7	19	52.7
	8–14	16	44.4
	15–30	1	2.9
Number of additional hospital stay due to SSIs	≤ 7	7	19.4
	8–14	19	52.8
	15–30	8	22.2
	≥31	2	5.6

### Socio-demographic determinants of surgical site infection

In this study, socio demographic variable had no significant association with SSIs except age, those women age less than nineteen years were three times risk of developing surgical site infection as compare to those age range 20–34(OR = 3.5, 95%CI 1.17–10.01) (Table 4).

**Table 4:** Socio-demographic characteristics and surgical wound infection among women having obstetric surgery in Assela Teaching Hospital, April, 23, 2015 to September 5, 2015

Variable	Category	SSI		*Crude OR(95%CI)*
		Yes Number (%)	No Number (%)	
Age	≤ 19	5(13.9%)	18(52%)	3.453(1.18–10.00)
	20–34	23(63.9%)	286(82.2%)	1
	≥35	8(22.2%)	44(12.6%)	2.26(0.95–5.37)
Residence	Urban	17(47.2%)	149(42.8%)	1
	Rural	19(52.8%)	199(57.2%)	0.837(0.437–1.66)
Ethnicity	Oromo	24(66.7%)	235(67.5%)	1
	Amhara	8(22.2%)	54(15.5%)	1.45(0.618–3.404)
	Tigrie	2(5.6%)	30(8.6%)	0.653(0.147–2.901)
	Guragie	1(2.8%)	19(5.5%)	0.515(0.066–4.020)
	Other	1(2.8%)	10(2.9%)	0.979(0.120–7.981)
Religion	Orthodox	17(47.2%)	119(34.2%)	1.500(0.729–3.088)
	Protestant	3(8.3%)	58(16.7%)	0.543(0.153–1.931
	Muslim	16(44.4%)	168(48.3%)	1
	Other	0	3(0.9%)	0.000
Occupation	House Wife	26(72.2%)	199(57.2%)	2.047(0.815–5.14)
	Civil Servant	6(16.7%)	94(27%)	1
	Farmer	0	3(0.9%)	0.000(0.000)
	Merchant	4(11.1%)	52(14.9%)	1.205(0.325–4.465)
Education Status	Illiterate	10(27.8%)	67(19.3%)	1.727(0.64–4.783)
	Read and Write only	2(5.6%)	51(14.7%)	0.454(0.091–2.270)
	Grade 1–8	7(19.4%)	61(17.5%)	1.328(0.442–3.985)
	Grade 9–12	10(27.8%)	88(25.3%)	1.315(0.478–3.617)
	Above Grade 12	7(19.4%)	81(23.3%)	1
Month Income	≤ 1000	17(47.2%)	158(45.4%)	0.890(0.400–1.983)
	1001–3999	8(22.2%)	99(28.4%)	0.669(0.257–1.736)
	≥4000	11(30.6%)	91(26.1%)	1

### Association of surgical site infection and obstetric variable

In the present study there was a statistically significant association between gestation age and SSIs, preterm gestation age mothers were four times more likely to develop SSIs as compared to those mothers gestation age was term OR = 4.225; 95%CI (1.254–14.238). The duration of labor had statistically significant association with SSIs, those duration of labor more than twenty four hours were two times more likely to develop SSIs as compared to those mother duration of labor less than twenty four hours OR = 2.219; 95% CI (1.054–4.670). Similarly, duration of membrane rupture had statistically significant association with SSIs, duration of greater than twelve hours were six times more likely to develop SSIs as compared to those mother duration of membrane rupture less than twelve hours OR = 5.991; 95% CI (2.757–13.022). The presence of chorioamnionitis had statistically significant association with surgical site infection, those mother who had chorioamnionitis were ten times likely to develop SSIs as compared to those mother who had no chorioamnionitis (OR = 9.743, 95%CI 3.077–30.848). Parity and ANC follow up had no statistically significant association with SSIs in this study (Table 5).

**Table 5:** Association of surgical site infection and obstetric variable among women having obstetric surgery in Assela Teaching Hospital, April, 23, 2015 to September 5, 2015

Variable	Category	SSI		*Crude OR(95%CI)*
		Yes number (%)	No number (%)	
Parity	I	11(30.6%)	101(29%)	1.343(0.595–3.034)
	II-IV	15(41.7%)	185(53.2%)	1
	≥ V	10(27.8%)	62(17.8%)	1.989(0.85–4.655)
ANC Follow Up	Yes	26(72.2%)	284(81.6%)	1
	No	10(27.8%)	64(18.4%)	1.707(0.784–3.716)
Gestation Age	Preterm	4(11.1%)	10(2.9%)	4.225(1.254–14.238)
	Term	32(88.9%)	338(97.1%)	1
Duration of Labor	≤ 24 h	24(66.7%)	284(81.6%)	1
	≥25 h	12(33.3%)	64(18.4%)	2.219(1.054–4.670)
Duration Rupture of Membrane	≤12 h	23(63.9%)	318(91.4%)	1
	≥12 h	13(36.1%)	30(8.6%)	5.991(2.757–13.022)
Chorioamnionitis	Yes	6(16.7%)	7(2%)	9.743(3.077–30.848)
	No	30(83.3%)	341(98%)	1
Meconium	Grade III	12(33.3%)	83(23.9%)	1.596(0.765–3.33)
	No	24(66.7%)	265(76.1%)	1
	Total	36	348	

### Surgical characteristics and surgical site infection

According to the present study the type of incision had statistically significant association with SSIs. Study subjects underwent vertical skin incision were four times more likely to develop SSIs than transverse skin incision (OR = 4, 95%CI 1.709–13.322). Individuals who had taken ceftriaxone and Metrinadazole were seven times more likely to develop SSIs than Ampicillin (OR = 7.5, 95%CI 3.115–18.004). The pre operation hematocrit had statistically significant association with SSIs, those women who had hematocrit value less than twenty one were six times more likely to develop SSIs as compared to those who had hematocrit value greater than thirty three (OR = 6.4,95%CI1.021–40.137). Moreover, those women who received transfused blood were six times more likely to develop SSIs as compared to women did not receive transfused blood (OR = 6.75,95%CI 2.471–18.489). Women underwent abdominal hysterectomy were eight times more likely to develop SSIs as compared women underwent cesarean section (OR = 7.9,95%CI 1.698–36.960). The pre-existing medical illness had also statistically significant association with SSIs; individuals with diabetic mellitus 3. 7 times more likely to develop SSIs as compared to those who had no pre-existing medical illness (OR = 3.7, 95%CI 1.112–12.519). The type of anesthesia, duration admission of prophylactic antibiotic, prior cesarean section, circumstance of surgery and intraoperative blood loss had no statistically significant association with SSIs (Table 6).

**Table 6:** Association of surgical site infection and surgical variable among women having obstetric surgery in Assela Teaching Hospital, April, 23, 2015 to September 5, 2015

Variable	Category	SSI		Crude OR(95%CI)
		Yes Number (%)	No Number (%)	
Type of Anesthesia	Spinal	30(83.3%)	306(87.9%)	1
	General	6(16.7%)	42(12.1%)	1.457(0.573,3.708)
Prior Cesarean section scar	Yes	4(11.1%)	38(10.9%)	1.020(0.342,3.041)
	No	32(88.9%)	310(89.1%)	1
Circumstance of Surgery	Emergency	31(86.1%)	313(89.9%)	0.693(0.253,1.898)
	Elective	5(13.9%)	35(10.1%)	1
Type of Operation	Cesarean section	32(88.9%)	338(97.1%)	1
	Abdominal hysterectomy	3(8.3%)	6(1.7%)	7.922(1.698,36.960)
	Uterine Repair	1(2.8%)	4(1.1%)	2.641(0.286,24.340)
Type of Skin Incision	Vertical Incisions	6(16.7%)	14(4%)	4.771(1.709,13.322)
	Transverse Incision	30(83.3%)	334(96%)	1
Time of antibiotic admission	With 30 min before surgery	22(61.1%)	252(72.4%)	1
	After operation	14(38.9%)	96(27.6%)	0.662(0.138,2.624)
Type of antibiotic	Ampicillin	26(72.2%)	331(95.1%)	1
	Ceftriaxone &Metrindazole	10(27.8%)	17(4.9%)	7.489(3.115,18.004)
Intraoperative blood loss	Less than 1000	35(97.2%)	344(98.9%)	1
	Greater than 1000	1(2.8%)	4(1.1%)	2.457(0.267,22.595)
Perioperative Blood Transfusion	Yes	7(19.4%)	12(3.4%)	6.759(2.471,18.489)
	No	29(80.6%)	336(96.6%)	1
Acute /Chronic medical problem	UTI	0	12(3.4%)	0.000
	Pneumonia	1(2.8%)	8(2.3%)	1.282(0.155,10.642
	HIV/AIDS	1(2.8%)	3(0.9%)	3.420(0.344,34.021
	Diabetes mellitus	4(11.1%)	11(3.2%)	3.731(1.112,12.519)
	Other	3(8.3%)	37(10.6%)	0.832(0.240,2.878)
	No known illness	27(75%)	277(79.6%)	1

### Multivariate logistic regression analysis of risk factors of surgical site infection

The outcome of the final backward multiple logistic regression models indicated that preoperative hematocrit, type of antibiotic, type of skin incision,duration of labor, type of operation and gestation age were dropped from the final model. However, age less than 19 years had statistical significant association with SSIs. Study subjects aged less than 19 years were 3.8 times more likely to develop SSIs when compared to age 20–34 years. Women who had chorioamnionitis were 9.1 times more likely to develop SSIs than those had no chorioamnionitis. Moreover, duration of rupture membrane greater than 12 h had also significant association with SSIs. Women who received perioperative transfused blood were 3.8 times more like to develop SSIs as compared to those who did not receive transfused blood. Women with diabetic mellitus were 3.1times more likely to develop SSIs when compared to women who had no any pre-existing medical illness in the present study (Table 7).

**Table 7:** Crude and adjusted associations of risk factors surgical site infection among women having obstetric surgery in Assela Teaching Hospital, April, 23, 2015 to September 5, 2015

Variable	Category	SSI		COR(95%CI)	AOR (95% CI)
		Yes Number (%)	No Number (%)		
Age	≤ 19	5(13.9%)	18(52%)	3.453(1.18,10.00)	3.81(1.05,13.83)
	20–34	23(63.9%)	286(82.2%)	1.0	1.0
	≥35	8(22.2%)	44(12.6%)	2.26(0.95–5.37)	2.13(0.79,5.73)
Duration Rupture of Membrane	≤12 h	23(63.9%)	318(91.4%)	1.0	1.0
	≥12 h	13(36.1%)	30(8.6%)	5.99(2.75–13.022)	3.77(1.53,9.31)
Chorioamnionitis	Yes	6(16.7%)	7(2%)	9.74(3.08–30.84)	9.05(2.35,34.83)
	No	30(83.3%)	341(98%)	1.0	1.0
Perioperative Blood Transfusion	Yes	7(19.4%)	12(3.4%)	6.76(2.47,8.49)	7.78(2.37,25.54)
	No	29(80.6%)	336(96.6%)	1	1.0
Acute /Chronic medical problem	UTI	0	12(3.4%)	0.000	0.000
	Pneumonia	1(2.8%)	8(2.3%)	1.28(0.15,10.64)	0.50(0.02,9.18)
	HIV/AIDS	1(2.8%)	3(0.9%)	3.42(0.34,34.02)	3.14(0.25,39.0)
	Diabetes mellitus	4(11.1%)	11(3.2%)	3.73(1.11,12.52)	5.41(1.45,20.11)
	Other	3(8.3%)	37(10.6%)	0.83(0.24,2.88)	0.46(0.11,1.94)
	No	27(75%)	277(79.6%)	1.0	1.0

## Discussion

SSIs represent a burden to the health care system and patient, mainly attributable to the extended length of stay in hospital and additional treatment required. Consequently, strategies and intervention aimed at reducing the incidence of SSIs could provide cost-saving and improve the efficiency of the health care system. The rate of SSIs were lower when we compared our finding with different studies conducted in African countries but still higher than the studies conducted in developed countries [8, 9, 17–21].

The prevalence of SSIs was 36(9.4%) in this study. This finding was lower than the study done in Jimma University Specialized Hospital which was 11.4% [22]. The other studies conducted in Africa, like Nigeria and Tanzanian were 23.6%, and 24% respectively [16, 23]. The prevalence of SSI in this study was lower compared to studies done in different hospitals in African countries. This difference might be due to lost to follow up the development of SSI after discharge.

Majority of the infections were confined to the incision site (94.5%) and the rest involved the organ/spaces accessed during operation in this study.

With regard to age, age had statistically significant association with SSIs *p* = 0.04. This was in line with previous report finding(OR = 2.1) [19]; women age less than 19 years had 3.8 more likely to develop SSIs as compared to age 20–34 years (OR = 3.81 95% CI 1.05, 13.83). An increased risk of SSIs in younger women had been reported in age less than twenty years (OR = 1.9) [20], but the study conducted in Jimma University Specialized Hospital indicated that age had no significant association with SSIs [8].This might be due to labor abnormality, poor nutrition (such as anemia), decreased immune completeness, urinary tract infection which lead to premature rupture membrane.

In this study prolonged rupture of membrane, greater than twelve hour were predicator of SSIs with *p* = 0.00. This was similar with the previous studies [8, 16, 17, 20, 21]; women with prolonged rupture of membrane(greater than twelve hour)had 3.8 times more likely to develop SSIs as compared to duration rupture of membrane less than twelve hour (OR = 3.771 95% CI 1.53,9.31).Normally during pregnancy,cervical mucus and amniotic fluid serve as barrier to infection. However if the membrane is ruptured, this protective effect is gradually reduced over as amniotic fluid become no longer sterile. Thus, it was though that the non-sterile amniotic fluid might act as a transport medium by which bacteria got the chance to contact with uterus and skin incision and this might resulted in chorioamnionitis.

In our study chorioamnionitis had statistically significant association with SSIs with *p* = 0.00; women with chorioamnionitis had 9.7 times more likely to develop SSIs when compared to those women who had no chorioamnionitis (OR = 9.74 95% CI 2.35, 34.83), and this had been observed in the previous studies [1, 8, 16–18, 21–26]. The gestational age andduration of labor had no association with SSIs in this study, this was in line with previous study findings [1–4, 7, 8, 12, 13, 16–18, 21–28]. In contrast to previous study done in Jimma University Specialized Hospital [8], no significant association was observed among ANC follow up and surgical site infection in this study.

Intake of Preoperative transfused blood was predictor of SSIs with *p* = 0.00; women who had received preoperative blood transfusion were 7.7times more likely to develop SSIs as compared to those who had no received blood transfusion (OR = 7.78 95% CI 2.37,25.54),and this had been observed in the previous study [20]. However there was no scientific basis for withholding necessary blood products from surgical patients as a means of either incisional or organ/space SSI risk reduction.

In our study diabetes mellitus was found to be predicator of SSIs with *p* = 0.01; women with diabetic mellitus had 5.4 times more likely to develop SSIs as compared to those with non-diabetic mellitus and this was in line with the previous study [21]. This could be due to abnormalities in cell-mediated immunity and phagocyte function associated with hyperglycemia, as well as diminished vascular supply to tissue and increased rate of colonization of *S. aureus* in the skin folds.

## Conclusions

In the present study the rates of SSIs was high. Women who were age less than 19, prolonged rupture of membrane, chorioamnionitis, preoperative blood transfusion and diabetes mellitus had been found predictors of SSIs. These five independent risk factors should be considered when establishing strategies for SSIs prevention and surveillance. Therefore,

Obstetric ward of Assela teaching referral hospital are encouraged to use properly the WHO surgical safety checklist and examine how to sensibly integrate these essential safety steps into their normal operative workflow. Prophylactic antibiotic administration should be provided within one hour before the surgical incision or within two hours if the patient is receiving vancomycin or floroquinolones.

Health information should be provided on ANC follow up, postnatal follow up and early marriage.

This study could be subjected to cross sectional study design bias and it was taken as the limitation of this study.

## Abbreviations

CDC’s: Centers for Disease Control and Prevention’s
NHSN: National Healthcare Safety Network
SSIs: Surgical Site Infections
